# Purification of Monoclonal Antibodies Using Chromatographic Methods: Increasing Purity and Recovery

**DOI:** 10.34172/apb.43967

**Published:** 2025-01-05

**Authors:** Elnazalsadat Jafarzadeh Chehraghi, Parvin Akbarzadehlaleh, Karim Shamsasenjan

**Affiliations:** ^1^Department of Pharmaceutical Biotechnology, Faculty of Pharmacy, Tabriz University of Medical Sciences, Tabriz, Iran; ^2^Department of Biochemistry, Faculty of Medicine, Tabriz University of Medical Sciences, Tabriz, Iran

**Keywords:** Monoclonal antibody, Purification, Bio-Separation, Chromatography, PEGylated monoclonal antibody

## Abstract

Monoclonal antibodies (mAbs) have gained increasing significance in biopharmaceutical research and production because of their precise targeting and therapeutic potential. The purification of mAbs is a crucial stage in production, ensuring the elimination of impurities for a top-quality, safe, and efficient final product. Chromatographic methods including affinity chromatography, ion exchange chromatography, and hydrophobic interaction chromatography (HIC) are commonly utilized to selectively capture and purify mAbs from complex mixtures by exploiting their unique properties, such as antigen-binding specificity or their surface charge and hydrophobicity. This review provides an overview of the current chromatographic techniques for mAbs purification, and to this aim delves into recent advancements and emerging trends in mAb purification, including the application of multi-modal ligands, membrane adsorbers, and continuous processing. These innovations aim to enhance efficiency, selectivity, and reliability while reducing processing time and costs, ultimately contributing to the development of safe and effective mAb-based therapies. Emphasis is placed on the necessity of choosing suitable methods based on the unique properties of the mAb and the desired quality attributes of the end product.

## Introduction

 In the production process of bio-pharmaceuticals, downstream processing has equal economic value and sensitivity as upstream processing. Among the bio-pharmaceuticals, monoclonal ant`ibodies (mAbs) are well known for their impact on research, diagnosis, and clinical applications.^1^

 Since 1975 when Kohler and Milstein introduced the hybridoma technology to produce antibodies, the downstream processing is still challenging to find a method to achieve higher purity and yield, besides being economically reasonable.^2,3^ Using recombinant DNA technology, mAbs have been successfully reconstructed through genetic engineering to produce chimeric, humanized, and fully human antibodies.^4^

 Bio-separation and purification of mAbs have been studied for many years and various chromatographic and non-chromatographic techniques have been reported in this regard. Generally, the CIPP strategy includes capture, intermediate purification, and polishing steps. In each step, based on the physicochemical properties of the target monoclonal antibody, the accompanying impurities, and the desired final purity, specific methods can be employed.^5^ In the capture step, the aim is to remove excess volume of buffer or media and isolate the proteases. In the two following steps, the target product separates from bulk impurities and then purifies from the remaining protein-based impurities. In the purification of mAbs, various chromatographic methods have been applied. Selecting proper and effective strategies in the capture phase may even cause moving directly to the polishing step which on a large scale is highly desirable.^6-8^

**Figure 1:**
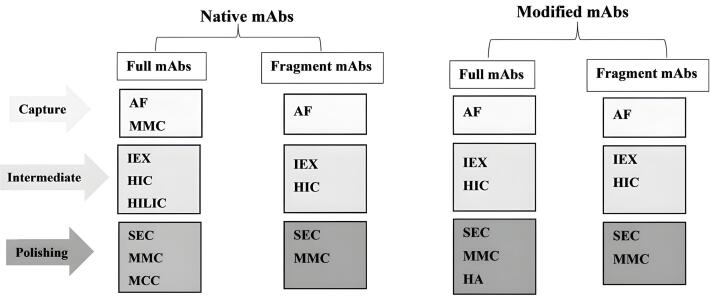
Comparison of purification steps in CIPP strategy between native (full & fragment) and modified (full & fragment) monoclonal antibodies

 This review summarizes the various chromatographic techniques applied in separating and purifying mAbs and reports the content in categorized titles for native and modified (e.g., PEGylated) mAbs. For better understanding, the methods used to purify native and modified mAbs are also reviewed in the form of four general categories: Native-full mAbs, Native fragment mAbs, Modified-full mAbs, and Modified fragment mAbs. In each of these four categories, different specific methods are used during the three phases of the CIPP process (capture, intermediaries, and polishing). These methods may vary depending on the physicochemical properties of the target mAb and the level of purity required for the target protein, as well as the company’s purification strategy (Figure 1). These methods are described in detail below.

## Separation of native monoclonal antibodies

###  Native full monoclonal antibodies 

 In vivo, administration of antibodies presents challenges due to contaminants such as DNA, viruses, pyrogens, and leachate. When producing biotherapeutics, 50-80% of the manufacturing costs are spent on further processing. The separation process includes three steps: enzymatic enrichment to remove impurities, intermediate purification to achieve 40%-90% purity, and polishing to separate rare impurities to near 100% purity.^9,10^ Affinity-based methods are commonly used in most monoclonal antibody purification processes due to their high specificity.

###  Affinity chromatography (AF)

 Affinity chromatography, introduced in 1968, purifies mAbs using protein A and protein G ligands due to their high affinity and specificity for the Fc-region. Protein A/G affinity chromatography offers quick separation, but harsh conditions and expensive resin can impact delicate antibodies. Protein A/G is immobilized on agarose and placed on a chromatographic column.^11,12^ This method can purify biomolecules based on their biological properties, ensuring high purity levels above 95% in one step. It involves binding mAbs to immobilized ligands, washing off unbound materials, and recovering bound antibodies through changing buffer conditions, resulting in high purity levels.^13,14^ In the following, almost all the research on the purification of mAbs by affinity chromatography method has been presented in chronological order.

 In 1991, Kyung’s study examined the impact of sample application velocity, sample volume changes, and column dimensions on the elution concentration profile of a product. The results showed that changes in sample velocity did not significantly affect the elution profile, and the elution concentration was independent of IgG concentration. The study also highlighted the dynamics of immunoglobulin separation using protein A affinity chromatography, with affinity chromatography A being the most effective technique for monoclonal antibody isolation.^15^ In 1997, Linda O. and colleagues studied the effect of different elution solvents on the conformation of mAbs against the human epidermal growth factor receptor 2 receptor (sHER2). They found that glycine at pH 2.9 maintained antibody structure, while 7 M urea partially unfolded antibodies. Complete unfolding occurred with 6 M guanidine HCl at pH 4.0. Refolded antibodies formed antigen-antibody complexes, and immunoaffinity chromatography revealed elution differences, highlighting the impact of elution solvents on mAbs’ recovery, refolding, and antigen recognition.^16^ Tugcu et al examined the optimization of resins for monoclonal antibody purification, focusing on available options such as protein A, protein A mimetic, mixed-mode interaction resins, and ion exchangers. Finally, they found that the dynamic binding capacity of these resins depends on the charging rate. A three-step purification process using these resins was found to be the most effective in terms of productivity. The research found that Mab Select, followed by UNO sphere Q and UNO sphere S, provided the most effective purification process in terms of productivity.^17^

 During protein A affinity chromatography, differences in binding capacities between antibodies and Fc fusion proteins were reported in a paper by Ghose et al in 2005. The underlying reason for the differences observed in dynamic binding capacities between different antibodies and Fc-fusion proteins is explored in this paper using various test molecules and commercially available protein A stationary phases. Based on the results obtained, dynamic binding capacity varies between 15 and 50 g of antibody per liter of resin, depending on the flow rate, antibody type to be purified, and protein A matrix used.^18^ Several studies have focused on separating therapeutic proteins like mAbs from plasma and serum. Research has shown that affinity chromatography is effective in this separation process. Developing new ligands in affinity chromatography and optimizing existing ligands will be critical to achieving higher yield and purity of mAbs. Furthermore, the use of multimodal ligands and the combination of affinity chromatography with other purification techniques can further improve the efficiency of the purification process.^19^ A study conducted in 2016 by L. Chen discussed the purification of mAbs produced by transgenic rice callus using the 2A self-cleavage peptide. Protein A affinity chromatography method effectively removed impurities, resulting in high-quality mAb. Purified antibody tested for binding affinity with hVEGF antigen using ELISA, and showed similarity to commercial Bevacizumab. Their applied purification method was successful in producing bioactive mAb with binding properties similar to the commercial version.^20^ J. Scheffel’s 2020 study describes a new purification process that combines the advantages of protein A resin, which provides excellent selectivity and recovery of 88%-99%, with a particularly mild elution step similar to ion-exchange chromatography for functional antibodies. MAbs bound to a tetrameric ZCa resin can be released by removing calcium ions with a chelator. Citrate buffer and EDTA serve as effective chelators.^21^ In 2020, a protocol was developed for purifying bevacizumab with affinity chromatography. The protocol involves attaching a monoclonal antibody with a strong binding to the VEGF protein in agarose. This binding allows for the isolation and purification of bevacizumab from cell culture supernatants or other sources, resulting in high purity and activity.^22^ A 2020 study by Akhtar evaluated protein A affinity chromatography for purifying a recombinant monoclonal antibody from cell culture using methods like SDS-PAGE, cation exchange chromatography, size exclusion chromatography (SEC), and reversed phased-reduced chromatography, achieving a 99% removal of impurities.^23^

 Another study in 2020 by Valsecchi explored the purification methods employed for emicizumab. The authors utilized a protein A affinity chromatography. This step was crucial in removing impurities and contaminants from the sample and isolating the target protein. This purification method ensured the quality and purity of the emicizumab used in this study.^24^ A recently published study in 2023 presented their findings on the production and assessment of atezolizumab, a newly approved monoclonal antibody that targets the immune checkpoint PD-L1, within plants. This study shows the viability of utilizing plants as a platform to create biologically active atezolizumab with comparable functionalities to commercially available versions. Atezolizumab was purified from crude extracts in a bioreactor using protein A affinity chromatography, as seen in the purification of plant-produced atezolizumab from *Nicotiana benthamiana* proteins.^25^

###  Ion exchange chromatography (IEX) 

 After using affinity chromatography in monoclonal antibody purification, additional methods are needed to obtain pure and high-quality antibodies. Ion exchange chromatography is a commonly used technique after protein-A chromatography due to its speed and cost-effectiveness. This method is often used for antibody fragments without Fc domains, making protein-A chromatography unnecessary.^26^ In 1975, ion exchange chromatography was developed by Small, Stevens, and Bauman. Later Gjerde et al reported an anion chromatography method in 1979, followed by a cation chromatography method in 1980.^27^ Ion exchangers are classified as anion and cation exchange chromatography (strong or weak) depending on their capacity to keep a charge within a specified pH range. The pI value of the target protein is critical for proper ion exchange chromatography.^28,29^ Anion exchange chromatography draws negatively charged proteins to positively charged supports containing functional groups such as diethyl amino ethyl (DEAE) and trimethylammonium ethyl (TMAE). Common anion exchangers include Fractogel EMD DEAE(M), Q Hyper D, and Q Sepharose Fast Flow. Cation exchange chromatography binds positively charged proteins to negatively charged solid supports.^30^ Cation exchange chromatography attracts positively charged proteins using a stationary phase with negatively charged groups like sulfonic or carboxylic acids; common resins include SP Sepharose and CM Sepharose. The binding of positively charged proteins is influenced by pH, ionic strength, and the protein’s net charge. Elution in ion exchange chromatography typically involves changing the salt or pH of the buffer gradually.^31^ A monoclonal antibody (mAb) purification process typically involves both cation exchange and anion exchange chromatography steps. However, it is important to consider several factors when designing such processes, including the mobile phase (pH, salt concentration), stationary (immobile) phase (type of ion exchange group, ion exchange capacity, particle diameter, pore structure, pore size distribution, the base matrix properties), column parameters (length, diameter) and operational variables (flow rate, gradient volume, sample loading).^32^ The weak partitioning chromatography (WPC) mode in ion exchange chromatography (IEX) has gained popularity for better impurity clearance and pH and conductivity optimization. A 2008 study discussed an advanced IEX step for mAb purification using WPC, which enhances product purity and can handle higher loads while maintaining recovery rates.^33^ Using anion-exchange chromatography, an orthogonal analytical technique has the potential for product quality assessment of monoclonal antibody therapeutics. According to a study done in 2011, anion exchange chromatography is used to separate oxidized mAbs.^34^ Using a lumped kinetic model with a pH and salt dependence included in the Langmuir adsorption equilibrium model in 2012, the separation of monoclonal antibody charge variants was successfully modeled using the lumped kinetic model.^35^ Different phase combinations affect retention, selectivity, and efficiency in ion exchange chromatography. The whole phase system must be considered for method development. Larger packed column particles resulted in lower retention with the same ion-exchange group. Column retention, efficiency, and selectivity varied significantly based on the elution mode. Additionally, short, narrow bore ion exchange columns enable 4–6 minute separations of intact and partially digested antibodies.^36^

 A technique called cross-interaction chromatography was introduced in a study in 2009. It quickly identifies soluble monoclonal antibody candidates. Researchers determine antibody solubility by coupling antibodies to an NHS-activated chromatography resin and analyzing their retention times. Adding this tool to the suite of tools available for characterizing antibody candidates will improve the selection of candidates for high-concentration antibody formulations. The study shows that antibodies prone to precipitation have longer retention times. This screening method successfully identifies therapeutic antibody candidates with high solubility.^37^ In 2014, a study optimized the separation of 10 monoclonal antibody charge variants using cation exchange chromatography. The Milli-Q Purification System column was used, and the sample was eluted with a salt gradient and pH gradient. The study concluded that generic conditions can be used for the characterization of mAbs with a wide range of pI values, with a strong cation exchange column and salt gradient CEX being considered a generic multi-product approach.^38^ A 2020 study investigated the optimization of monoclonal antibody charge variant purification using cation exchange chromatography under different conditions (different resins, elution mode, gradient slope, and flow rate). The target mAb’s purity and recovery were used to determine the desired outcome. As a result, only the resin with a small particle diameter (15 µm) Nano SP-15L was able to achieve nearly 100% purity with a 56.5% recovery.^39^

 Another research conducted in 2023 delves into the purification and biotransformation of mAbs (trastuzumab and pertuzumab) in individuals with breast cancer. The investigators employed affinity enrichment utilizing Affimer (affinity enrichment) reagents and ion-exchange chromatography to separate and assess the antibodies from blood samples. Moreover, they replicated in vitro biotransformation by exposing the antibodies to 37 °C for durations of 1 and 2 weeks. The primary objective of the study was to elucidate the effects of biotransformation on the therapeutic effectiveness and safety profile of these antibodies, which have the potential to impact patient responses to treatment.^40^

###  Hydrophobic interaction chromatography (HIC)

 HIC is a commonly employed technique for the purification of mAbs. HIC often have been employed as a polishing step in monoclonal antibody purification processes, as it offers an orthogonal separation mechanism to other chromatography techniques. HIC can remove product-related impurities and process contaminants, resulting in an ultra-pure monoclonal antibody product.^41^ The separation in HIC is based on the hydrophobic surface characteristics of the proteins in the sample. When a salt concentration gradient is applied, molecules with higher surface hydrophobicity elute; nevertheless, in aqueous conditions with a high salt concentration in the buffer, they preferentially attach to HIC resins containing ligands such as phenyl and butyl.^42^

 The main advantage of HIC over other chromatography techniques is its ability to separate under mild, non-denaturing conditions. It can also eliminate various impurities like host cell proteins, host cell DNA, and leached protein A making it a good candidate for being used after ion exchange chromatography as a polishing step.^43^ However, HIC has some disadvantages, such as the requirement for high salt concentration, which might cause protein precipitation, and the need to carefully adjust the mobile phase condition. Moreover, it has a relatively lower yield compared to other chromatography steps.^42^ A study by Chen and Cramer in 2007 assessed protein adsorption in hydrophobic interaction chromatography by analyzing isotherms on different HIC resin systems. In this study, non-linear binding isotherms, are observed in HIC, and their consequences in protein purification have been discussed. Non-linear binding isotherms can cause a lack of resolution and efficiency in HIC, but careful selection of operating conditions can reduce these effects.^44^ HIC is regarded as the definitive method for isolating hydrophobic variants of mAbs and their subunits, as well as for examining the diverse populations of cysteine-linked antibody-drug conjugates (ADCs) molecules.^45^ The HIC method is utilized for obtaining ultra-pure antibodies. By using hydrophobic adsorbents such as Toyopearl® Phenyl 650 M in the presence of ammonium sulfate in the polishing step, it can produce 99.9% pure antibodies with 96.2% recovery.^46^ Generally, utilizing HIC for monoclonal antibody purification proves it to be a practical, effective, and versatile method within the biopharmaceutical sector. Ongoing research focuses on novel sorbents, optimized conditions, and new applications to enhance the value and impact of this technique. Overall, the continued advancement of HIC in the purification of mAbs is crucial for improving the quality and availability of therapeutic proteins.

###  Hydrophilic interaction liquid chromatography (HILIC)

 Regarding the purification of mAbs, compared to traditional reversed-phase liquid chromatography (RP-LC), HILIC has numerous advantages such as preservation of polar compounds, orthogonal selectivity, increased sensitivity, and reduced column back pressure. However, this method has some challenges, especially in terms of the complications of retention behavior and the incomplete description of retention mechanisms. Still, despite these challenges, HILIC offers a main tool in the isolation and identification of polar compounds.^47,48^ In 2017, a method for the separation and analysis of recombinant mAbs via HILIC was introduced. The study demonstrates a strategy to optimize HILIC parameters for different mAb types and an ADC using seven FDA and EMA-approved mAbs with different isoelectric points (pI) and different hydrophobic properties. The procedure involves sample digestion with the enzyme called IdeS and chemical reduction followed by HILIC separation to efficiently identify hydrophilic variants (glyco-variants) for protein-level analysis.^47^ Another study presented a method for analyzing by-products in monoclonal antibody mixtures using a C18 wide-pore protein trap column and HILIC. This method was designed to help greatly in ensuring the strength and quality of the mAb quickly and efficiently. The method is very quick and efficient and enables rapid evaluation of multiple samples. It can be useful for high molecular weight or highly hydrophilic excipients that challenge conventional detection methods.^49^ A 2022 study highlights the significance of glycosylation in monoclonal antibody therapies, demonstrating the effectiveness of the hydrophilic interaction liquid chromatography-mass spectrometry (HILIC-MS) method in analyzing protein glycosylation patterns, which affect the potency and longevity of mAbs and optimizing antibody production by identifying different glycan patterns.^50^

###  Size exclusion chromatography 

 SEC effectively purifies mAbs, typically after other chromatographic techniques. It can separate proteins, including mAbs, using a porous resin based on protein size relative to molecular weight. Separation occurs under gentle conditions using buffers with near-neutral pH and moderate salt content to reduce nonspecific interactions and keep proteins soluble. Depending on the molecular weight of the antibody, different resins such as agarose, dextran, or polyacrylamide are selected. SEC can operate in binding-elution or flow-through mode for efficient antibody production.^51^ The main advantage of SEC is the non-destructive separation of proteins. SEC effectively removes aggregates and impurities and delivers pure mAbs. Because of fasting and the efficiency of this method, it can be suitable for large-scale cleaning. However, SEC has protein size limitations and requires costly gel filtration matrices. Furthermore, SEC is less selective compared to techniques such as HIC. Therefore, SEC is often used as a polishing step along with other chromatographic methods to improve purification results for mAbs.^51,52^ Particle size and pore size of the phase can influence the efficiency of SEC, as lower efficiency was observed for samples with high contamination. A study conducted in 2016 showed that SEC can work with relatively large sample loadings (up to 4% column volume) and high flow rates (up to 120 cm/h at laboratory scale and 80 cm/h at laboratory scale and pilot scale. This allows for more efficient and better cleaning compared to traditional SEC with lower throughput.^53^ Farnan and colleagues conducted a study in 2009. Their project investigated how to double output power without degrading isolation. Sample injection was performed before washing the previous sample to minimize lag time and increase processing efficiency. A significant improvement of approximately 2-fold was obtained by coordinating the injection and diagnosis times. Based on their data, interlacing is a reliable method for enhancing throughput using size exclusion for mAbs.^54^ In 2010, a study developed a method for characterizing intact soluble monoclonal IgG1 antibody oligomers using mass spectrometry. High-performance size-exclusion chromatography successfully isolated monomeric and aggregated IgG fractions, while electrospray ionization time-of-flight (ESI-TOF) mass spectrometer analysis determined molecular weights, proving ESI-TOF MS a useful complement for identifying small oligomeric protein aggregates.^55^

 Another study conducted by Chakrabarti in 2018, discusses the use of analytical SEC in separating mAbs. The results showed that analytical SEC is a useful method in separating and characterizing mAbs. In addition, SEC is a gentle and non-destructive technique that does not require harsh conditions or denaturants, making it a useful tool for analyzing and purifying mAbs for research and development purposes.^51^

 Pavlu and his team studied three chromatography techniques in 1986 to isolate anti-VIII factor mAb from mouse ascites fluid. They also investigate the impact of (NH₄) ₂SO₄ precipitation on antibody purity before chromatography. The study found that HPLC techniques are nearly equally effective in purifying mAbs. One chromatographic step can yield a highly pure mAb if (NH₄)₂SO₄ is used initially. If (NH₄)₂SO₄ is not used, ascites can directly serve as the starting material for highly pure mAbs through two-step HPLC chromatography.^56^

 In 1985, Stanker developed a one-step method for purifying mouse mAbs using hydroxyapatite column chromatography. The immunoglobulin purified from this study was sufficiently pure to be used and reviewed in other hydroxyapatite chromatography studies. Furthermore, it appears that this method separates the monoclonal antibody from other antibodies regardless of the origin of the primary antibody. According to the results of the article, approximately 80-90% of antibodies were recovered, but this estimate is not accurate.^57^

###  Mixed-mode chromatography (MMC) and multi-column chromatography (MCC) 

 Multimodal and mixed-mode chromatography are chromatographic methods that use different interactions between the stationary phase and analytes. They have some differences. Multi-column chromatography uses multiple columns with different stationary solid phases to achieve high purity.^58^ Each column targets a specific impurity or molecule. The sample passes through each column in a specific order. This method can be time-consuming and requires more equipment. On the other hand, mixed-mode chromatography uses one column with ligands engaging in various interaction types. Both methods can achieve high selectivity and purity, but mixed-mode chromatography can be more cost-effective and requires less equipment.^59^

###  Mixed mode chromatography (multimodal chromatography) 

 In multimodal chromatography or mixed-mode chromatography, various interactions are integrated into a resin. It improves the purification of mAbs with better selectivity and capacity. This method is critical for removing aggregate and improving downstream efficiency. Implementation after protein A chromatography can streamline processes and increase productivity.^59-63^ Resins such as Capto Adhere, Capto MMC, HEA HyperCel, MEP HyperCel, and PPA HyperCel offer different release mechanisms.^61^ Controlling the pH gradient with these resins can result in unique separations.^64^ Capturing complex interactions and resin longevity is key to the economical purification of mAbs in large scale.^65^ In 2015, a study found that a mixed-mode chromatography column that utilizes the dual function of weak anion exchange/hydrophobic interaction can be very effective in purification. The aim is to separate proteins based on charge and hydrophobic properties for chromatographic protein purification. Using this method, the study was able to separate nine proteins within two hours. The results show that the MMC column is a promising alternative to two conventional columns for HIC and WAX modes.^66^ A 2017 study highlighted continuous countercurrent tangential chromatography (CCTC), a technique used for monoclonal antibody purification, for post-capture operations. Using a membrane system and mixed-mode chromatography resins, CCTC improves efficiency, yield, and productivity, with the potential for large-scale therapeutic antibody production due to its absence of discontinuous processes.^67^ A 2019 study examined the behavior of two multimodal cation exchange ligands, Capto MMC and Nuvia cPrime, in different solvents. How these ligands interact with proteins, particularly mAbs, has also been studied. Analysis of the conformational changes of the ligands in solution and upon binding to proteins contributed to the elucidation of protein-ligand interactions. The results suggest that the hydrogen bonding sites of the ligands experience less dehydration when binding to protein antibodies.^68^ Polyallylamine has been engendered with some hydrophobic groups to create new MMC resins. These resins can reduce HCP levels during mAb purification, they also can offer wide conductivity and can be used in one-step polishing. These resins have a high yield ( > 93%) and loading capacity (around 1000 mg/mL-resin) for mAb production, making them cost-effective and suitable for mAb manufacturing applications.^69^ In 2023, researchers investigated a new non-woven membrane for purifying mAbs. This membrane is distinguished by a polyvalent ligand, MPCA, coupled to a PGMA base. It aids cleaning by using charge differences and hydrophobic characteristics. The membrane has a high binding capacity and efficient purification, indicating its suitability for monoclonal antibody applications.^70^

###  Multi-column chromatography 

 The MCC technique involves using multiple columns connected in series, each with a specific function, to achieve a high degree of purification. In 2023, a continuous MCC method for purifying mAbs was developed. The process is divided into two phases: capture and polishing. The results showed significant improvements in productivity and efficiency as multiple columns could perform loading, washing, and regeneration simultaneously, resulting in shorter processing times and higher throughput.^58^ The 2024 article presents two monoclonal antibody purification methods: RC-PMCC and RC-BioSMB. The RC-PMCC method yielded 90% and had a productivity of 1010 g/L/day, while RC-BioSMB achieved 574 g/L. Both techniques successfully eliminated impurities and allowed bioreactor scaling to 2000 L.^71^ Multi-column displacement chromatography (MDC) separates the charge variants of antibodies using multiple columns of different solid phases, thus achieving high-resolution separation. MDC selectively elutes target molecules for higher purity and yield, outperforming ion exchange chromatography. However, the complexity of MDC requires careful optimization. In conclusion, MDC is an effective tool for purifying biotherapeutic molecules.^72^

###  Simulated moving bed chromatography (SMB)

 SMB chromatography, introduced in 1997, continuously purifies mAbs. It features a series of short resin beds that shift the inlet/exhaust positions to mimic resin movement. SMB outperforms traditional batch methods with higher productivity, purity, and less solvent consumption. Its flexible size adapts to production needs, making it a chameleon in various applications. In continuous operation, SMB increases adsorption and minimizes product dilution. SMB uses resins such as protein A and HIC types, including MacroPrep Methyl HIC. Although ideal for mAb purification, combining it with other methods increases purity and yield by targeting specific contaminants. SMB is a gem in biopharmaceuticals due to its expertise in mAb purification.^73-77^ It is crucial to highlight the distinction between SMB chromatography and MCC. SMB is a continuous multi-column process that has been used for industrial-scale separations for decades, utilizing a closed loop of multiple columns to achieve continuous operation and higher productivity. In contrast, MCC is a newer technique that uses multiple columns in parallel to increase throughput but does not necessarily involve continuous operation or a closed-loop configuration like SMB.^77^ A patent discussed utilizing SMB chromatography for purifying antibodies, with improved separation strategies and possible Raman spectroscopy. This patent pointed out the drawbacks of traditional batch mode chromatography which only utilizes 30%-50% of the column’s actual binding capacity, indicating that SMB could enhance column efficiency and capacity through the implementation of improved strategies.^75^ A review article published in 2016 addressed the new advances in separation technology suitable for uninterrupted downstream bioprocessing. This highlights the potential of SMB chromatography for continuous processing, which can increase adsorbent capacity and reduce product dilution.^78^

 SMB is employed in industry, such as Tarpon Biosystems Inc.’s BioSMB system, which allows for continuous feed loading and elution over several protein A columns. It is incorporated into the ASAP process and combines protein A affinity chromatography with CEX. SMB technology allows for continuous processing, decreasing time and expenses compared to traditional methods, increasing productivity, and lowering costs.^79^

 In 2019, Michelle Bahri published a paper on using SMB chromatography for monoclonal antibody purification. The article outlines SMB chromatography’s benefits over traditional methods, including better resin use, higher efficiency, and lower labor costs. It also discusses the theoretical and practical aspects of SMB chromatography, including the use of multiple short resin beds, movement of inlets and outlets, and zones in separation. The article also discusses its application in industrial settings like enzyme purification and monoclonal antibody production.^77^

###  Native fragmented monoclonal antibodies 

 Monoclonal antibody fragments, which lack Fc effector function but still have affinity similar to their original mAbs, are being studied as therapeutic agents. These fragments can be purified using certain chromatographic methods, which differ from full-length mAbs.^80^ Each fragment requires a specific purification protocol due to the absence of an Fc region. Affinity chromatography is commonly used for antibody purification, but newer resins offer alternatives for fragment capture. Intermediate purification and polishing steps can involve anion exchange or SEC based on different selectivity. Membrane-based techniques and precipitation have also been used for this purpose.^13,80-82^

###  Affinity chromatography 

 Affinity chromatography is one of the initial methods used to purify monoclonal fragments. Protein A, protein G, and protein L are types of affinity chromatography used for monoclonal antibody fragment purification. Protein A has a high affinity for IgG from many species. Protein G has broader binding specificity for IgG from different species.^82^ Protein L is used to purify antibody fragments, IgG, and other antibodies from various eukaryotic species. In affinity-tag-based chromatography, a tag is attached to the protein of interest, which is then used to purify the protein. In 1998, an analysis was conducted on HyHEL10 single-chain Fv fragments refolded in *E. coli* solubilized inclusion bodies. The purpose was to develop highly efficient refolding systems. The purification of a refolded scFv fragment achieved 150 µg/mL recovery using protein A affinity chromatography. This article concluded that protein A chromatography remains the most common method of antibody affinity purification at a manufacturing level.^82^ Fab or scFv fragments can be purified with a special tag during cloning. But, it’s possible to produce functional antibody fragments without tags. Affinity chromatography is the most efficient method for one-step purification. Human Fab fragments can be purified using anti-Fab antibodies or the columns with antigen-coupled. Protein L can purify Fabs with kappa light chains. Protein A or G affinity chromatography is commonly used to purify complete antibodies or Fc fragments. It can also bind to the V region of certain antibodies. Protein A affinity columns can still purify Fabs with either light chain when the heavy chain is from the V H 3 family.^83^ Another study published in 2010 described that protein A has a strong attraction to IgG antibodies, which allows for the purification of IgG, its fragments, and some subclasses. The purification process involves passing the cell culture supernatant over a column, where the antibodies bind and unwanted components flow through. Optional wash can remove non-specific impurities, and the product is eluted then at a different pH level.^13^

 A study conducted in 2013 discusses the purification of single-chain antibody fragments (scFv) using immobilized metal ion affinity chromatography (IMAC). The researchers explored the use of both discontinuous and continuous IMAC methods to purify scFv from E. coli lysates. Eventually, the authors concluded that both discontinuous and continuous IMAC methods are effective for purifying scFv proteins, with the choice of method depending on the specific needs of the researcher.^84^ A 2015 study described a method for purifying a monoclonal antibody fragment using a protein G affinity column. This method involves the use of a pH gradient elution strategy to achieve high purity and high yield of the antibody fragment. The authors showed that this method is effective for purifying various antibody fragments.^85^

 Because of their potent IgG binding, proteins A and G are recommended for antibody purification. The antibody subclass and purification objectives determine whether to use protein A or protein G. Because of its higher binding capacity, protein A is favored for large-scale procedures, but protein G is employed for low-affinity fragments; the fragment’s binding characteristics determine how effective protein G is. Choosing the best purification technique requires assessing each component’s properties.^86^

 Protein L affinity chromatography is commonly employed to capture antibody fragments that encompass the Fab component due to their inherent deficiency in affinity to protein A ligand. Protein L binds to the variable region of the kappa light chain and does not interfere with the antigen binding site. Protein L chromatography purifies antibody fragments with kappa light chains, including Fab fragments.^85^ A technique has been developed to modify antibody fragments so they can bind to protein L, a useful bacterial protein for antibody purification and detection. The method involves making changes to the variable light chain of the antibody fragment and has been successful with different types of antibodies. This modification could have many applications in research and diagnostics.^87^

 In 2017, Yang et al developed an scFv antibody targeting a furaltadone metabolite using phage display. The antibody was expressed in *E. coli* and purified using NiNTA affinity chromatography. The antibody achieved a purity of 97%, a yield of 20% and an amount of 29.1 mg. This research demonstrated the feasibility of phage display to generate antibodies against small molecules relevant to food safety and environmental monitoring.^88^ In 2021, the scFv antibody AK2 against CDK4 was generated using E. coli and purified using affinity chromatography. AK2 was amplified with a Ni2 + -NTA affinity column. A binding buffer containing 10 mM imidazole helped AK2 bind to the column more efficiently. A wash solution containing 40 mM imidazole removed nonspecifically bound proteins, while a 500 mM imidazole elution buffer isolated pure AK2. SDS-PAGE showed a single protein band of approximately 30 kD in the eluted fraction. Various tests confirmed the specificity of AK2 for CDK4. The authors believe that AK2 has the potential for cancer therapy.^89^

 In 2023, MCC was discussed in an article. First, SEC prevents aggregation, followed by a PCC column with affinity resin for further purification using protein L columns. This technique efficiently purifies Fab fragments from raw mixtures and eliminates impurities. The method increases the yield of purified Fab fragments while minimizing the purification steps needed.^90^

###  Size exclusion chromatography 

 Fragments like Fab and F(ab’)2 have advantages over whole MAbs as they reduce nonspecific binding. SEC can separate antibody aggregates, monomers, and degradation products. A 2009 study used acetonitrile, TFA, and formic acid as mobile phases for an SEC-MS method, which separates intact antibodies, heavy chains, light chains, and thioether-linked species without heating the column.^91^ The study used SEC and mass spectrometry to analyze antibody fragments. The research examines how different percentages of mobile phase components such as TFA, formic acid and acetonitrile affect monoclonal antibody fragment separation efficiency. Higher acetonitrile concentrations affected elution times but did not hinder the effective separation of Fab and Fc, in addition, the Zenix^TM^ SEC-300 column was found to be efficient for the mAb fragment separation.^92^ A 2020 study describes a method for analyzing aggregates and fragments in therapeutic mAbs using ammonium acetate-SEC buffer and a TOF mass spectrometer. This technique is suitable for studying aggregation mechanisms, degradation pathways, developing purification and formulation processes, and it can also lead to purification and formulation process development and support product assessment.^93^

###  Hydrophobic interaction membrane chromatography (HIMC)

 In 2014, a study examined the purification of EG2-hFc mAbs using HIMC instead of protein A affinity chromatography. The authors found that HIMC represents a valid alternative for the purification of chimeric heavy-chain mAbs.^94^ A new chromatography column, Thermo Scientific MAbPac HIC-10, was used to analyze mAbs and related substances. This column enabled high-resolution separation and could effectively distinguish mAbs from their related substances such as charge variants, aggregates and fragments. The researchers confirmed the usefulness of the column for the analysis of mAbs and their associated substances.^95^ Based on melting point measurements (using differential scanning calorimetry), it could be shown the conformational changes in antibodies on a HIC surface depend on the hydrophobicity of the stationary phase. Different mAb domains contribute to the overall binding strength and selectivity. HIC is a non-denaturing chromatographic technique that is useful for protein analysis.^96^

###  Ion exchange chromatography 

 A 2018 review examined purification methods for antibody fragments used in therapy and diagnostics. The review assessed various techniques, including affinity chromatography and ion exchange chromatography. It emphasized yield, purity, and recovery of fragments. Moreover, it noted the study of pure Fab fragments binding to non-affinity chromatography resins. The review pointed out that multimodal resins performed better at pH 5 and were salt-tolerant behavior.^97^

 In 2018, a study examined cation exchange chromatography to purify antibody fragments, focusing on reducing charge variants. The research found that the SP ImpRes resin combined with a salt and pH gradient showed very good effective separation performance, also the result can support the potential of this method for industrial production of therapeutic antibody fragments.^80^ In a study conducted in 2008, CHT-HPLC (multicolumn chromatography) was utilized to purify pepsin-derived monoclonal antibody fragments from murine ascitic fluids, demonstrating its effectiveness in purifying various classes of mAbs from crude preparations.^98^

###  Mixed mode chromatography (multimodal chromatography) 

 Multimodal chromatography is used to purify monoclonal antibody fragments such as Fab, Fc, and F(ab)2 fragments. In 2019, a new purification method for scFv was developed using hydrophobic charge induction chromatography and anion hydrophobic mixed-mode chromatography. This method resulted in high yield, purity, and reduced host cell proteins, with the scFv being selectively bound to the column due to hydrophobic and negatively charged spots.^99^

 In a study in 2022, a technique for studying the bispecific mAbs in multimodal chromatography is presented. It involves screening, mapping, and foot printing to understand different domain contributions. The Fab domain drives interactions with the resins, and both halves of the antibody bind to the resins. This technique can improve bio-manufacturability and bioprocess development.^100^ A 2023 article discusses a new method for quickly developing multimodal chromatography resins with unique selectivity for purifying biopharmaceuticals. A diverse virtual library of 100 Capto MMC ligand analogs was created, and 12 new ligands were synthesized and coupled to the Capto ImpRes agarose base matrix. The high-throughput screening tested the resin’s ability to bind six model proteins and improve separation resolution between mAbs, product-related impurities, Fab fragments, and high molecular weight aggregates.^101^

## Separation of engineered (modified) monoclonal antibodies

###  Engineered full monoclonal antibodies 

 Engineered mAbs can be classified into five categories: recombinant mAbs, bispecific antibodies, mAbs-drug conjugates, PEGylated mAbs.^102^ In the following section, some recent studies on this topic are discussed.

 Techniques like affinity chromatography and ion-exchange chromatography are often used in the purification of produced recombinant antibodies and mAbs, in addition, to reduce costs and improve efficiency, synthetic affinity ligands can be developed for antibody purification. These ligands are highly stable, selective, and inexpensive, making them ideal for large-scale manufacturing.^4^ An article published in 2010 discusses the expression, purification, and preparation of mAbs for equine interleukin-18. Equine interleukin-18 was produced and purified from *E. coli*. Its biological activity was similar to human IL-18. MAbs were produced by immunizing mice with equine interleukin-18. These antibodies recognized different epitopes and one of them could neutralize equine interleukin-18. In this study, equine interleukin-18 was successfully purified using nickel affinity gel column chromatography.^103^ From 1995 to 2005, Yamamoto and colleagues optimized the purification of recombinant mAbs using ion-exchange chromatography. They performed linear gradient elution experiments, obtained salt concentration and peak width data, and calculated the distribution coefficient and plate height. They determined optimized elution conditions based on buffer consumption, separation time, and salt concentration to provide a fast and effective method of purification.^32,104^ A study in 2015 discusses a process to purify and optimize recombinant mAbs from CHO cell culture supernatant. The process involves using mixed-mode resins and an anion exchange membrane. The yield and purity of the antibodies produced in the supernatant were 88% and 99.9% respectively. The purified fraction met regulatory specifications for aggregates, HCPs, and DNA levels. Contaminating proteins in the antibody fraction were identified using mass spectrometry to gain a better understanding of the behavior of HCPs.^105^ A study examined the adsorption of therapeutic recombinant mAbs on reversed-phase wide-pore stationary phases. Results showed that intact antibodies had higher adsorption compared to large biomolecules and recombinant mAbs of similar size, isoelectric point, or hydrophobicity. Also, a study tested two stationary phases, Phenomenex Aeris Wide pore, and Waters Acquity BEH300.^106^

 MAbs bearing engineered cysteine residues (termed THIOMAB^TM^ antibodies) enable the site-selective attachment of drugs, labels, or payloads for targeted delivery. Purification techniques like Disposable Cation Exchange Columns and Disposable Gel Filtration Columns are useful for the quick purification of conjugate reactions or the final formulation of conjugates.^107^

 A 2020 review article examines chromatographic methods for the purification and characterization of plant recombinant mAbs. Plants have the potential to produce recombinant proteins. The overview describes various downstream processes and chromatographic methods for purification, such as protein A affinity, ion exchange, hydrophobic interaction, and mixed-mode chromatography. In addition, the article examines analytical platforms using chromatographic techniques and detection systems to characterize plant mAbs at the protein, peptide, and glycan levels.^108^

 Chromatographic methods are commonly used to purify bispecific antibodies with a shared light chain and two different heavy chains. During purification specific antibodies concentrate and non-specific antibodies and contaminants are removed. Various chromatographic methods, such as protein A chromatography, HIC, and multimodal or mixed-mode chromatography, have been used for this purpose. A purification method using protein A chromatography has been conducted for the purification of bispecific antibodies. This technique guarantees a high level of purity ( > 90%) and yield ( > 85%), which is necessary for the production of bispecific antibody constructs.^109^ HIC is used for purifying bispecific antibodies from bioreactor supernatant. It can provide a good resolution between bispecific antibodies, monospecific immunoglobulins, and culture medium supplements. The purified proteins are analyzed for quality, functionality, and the presence of endotoxin and mouse DNA in the antibody preparations. This method reduces labor time, cost, protein loss, and risk of contamination compared to other purification methods.^110^ In a 2014 study, Hall et al employed CEX for the resolution of a bispecific antibody and the removal of a malformed mAb diabody species and aggregate products.^111^ Additionally, multimodal or mixed-mode chromatography has been used for the purification of fully human bispecific antibodies. This chromatographic method combines different types of interactions, including hydrophobic interaction, hydrogen bonding, and ionic interaction.^112^ A 2023 study explores the challenges of purifying bispecific antibody molecules using resins. Mixed-mode anion chromatography and hydroxyapatite chromatography were found effective for removing contaminants and achieving high purity and yield, successfully resolving bispecific antibodies, monospecific immunoglobulins, and culture medium supplements., showcasing its ability to purify BsAb.^113^

 Recent studies have shown that there are several approaches available for purifying ADCs including monoclonal ADCs. These include affinity chromatography and protein A-based chromatography.^52^ Additionally, methods like reversed phase HPLC and IEX, particularly CEX, play a crucial role in the analytical methods used for the physicochemical characterization of ADCs.^114,115^

 IEX chromatography is the main technique for purifying approved and commercialized PEGylated proteins including PEGylated mAbs. It helps to remove the small molecules and separate unreacted PEG molecules from the protein. IEX chromatography is widely used in downstream processes such as capture, intermediate, and polishing steps.^19^ Protein A chromatography is emphasized as a potent method for purifying mAbs, including PEGylated ones,^13^ offering precise and gentle purification for various antibody formats, complementing the effectiveness of IEX chromatography and PEG precipitation in purifying PEGylated antibodies.^74^ A 2021 review has examined ion exchange chromatography (IEX) and affinity chromatography (AF) to purify PEGylated mAbs. IEX is known for its resolution and ability to separate positional isomers of mono-PEGylated species. Affinity chromatography, particularly on Heparin Sepharose 6 FF, can be very effective for high yields and purity of PEGylated proteins. The study also examined on-column PEGylation as a technique that integrates PEGylation reaction, separation, and purification in just one step, resulting in higher yield in biopharmaceutical production.^19^

###  Hydroxyapatite chromatography (HA)

 Hydroxyapatite chromatography can be used as a popular method for isolating male mAbs. The advantages of this method, such as high selectivity, resolution, and robustness, have led to its application in various areas, including partial purification from protein A column eluates, selective desorption, high-throughput screening, one-step purification from crude murine ascitic fluids, and polishing to remove dimers, aggregates, and leached protein A. Therefore, it can be used both in laboratory and large-scale methods. However, challenges associated with this method encompass resin lot-to-lot variability, resin longevity, and viral clearance capacity.^116^ Hydroxyapatite chromatography will evolve with resin technology and process improvements. New multimodal resins like Capto MMC and Capto Adhere may enhance monoclonal antibody purification.^13^ A 1989 review focused on strategies for purifying mAbs. It emphasizes the benefits of hydroxyapatite chromatography over other methods, particularly its capacity to differentiate immunological constructs and eliminate Fab fragments from Fc contaminants. The publication highlighted the adaptability of hydroxyapatite chromatography to a range of applications, including purification of IgG, IgA, and IgM, as well as its effectiveness in eliminating antibody clusters, host cell proteins, and residual protein A from recombinant mAbs.^117^ A study examined HPLC with hydroxyapatite beads to separate mouse IgA and IgM antibodies. For optimization of the purification condition, the scientists use phosphate buffer gradient to elute distinct antibody forms. The final result shows that Hydroxyapatite chromatography can be an effective method to purify mAbs, remove impurities, and versatility in various applications.^118^ Another study has investigated the role of hydroxyapatite chromatography in removing antibody aggregates with polyethylene glycol (PEG) in a buffer. The data show that through PEG interaction, the retention time of aggregates increased on the HA column. The study shows that PEG incorporation improves hydroxyapatite chromatography’s efficiency in removing aggregates. In conclusion, the HA approach may reduce aggregate levels from over 60% to less than 0.1% and can be applied to a variety of antibody classes.^119^

 Ceramic hydroxyapatite (CHT) HPLC is another technique for purifying mAbs which uses hydroxyapatite microcrystals. This method employs metal chelation, phosphoryl cation exchange, and hydrogen bonding to interact with molecules. It can purify mAbs, proteins, and nucleic acids with high resolution, making it an ideal section for protein purification in both laboratory and industrial applications.^98^ A two-step technique for purifying mAbs that use CHT chromatography and membrane filtering has been presented, reducing operational time as compared to traditional procedures. The method eliminates 90.2% of mAbs during the elution phase, yielding a purity of more than 90% while retaining antigen-specific activity. This method avoids low pH elution, which can cause antibody aggregation and deactivation, and it eliminates the need for buffer exchange before chromatography, making it a more efficient and productive option alternative.^120^

 A 2019 study investigated the competitive binding of monoclonal antibody monomer-dimer combinations to CHT to better understand their adsorption behavior. The researchers discovered that the structure of both forms of CHT consisted of extended nanocrystals with varying pore diameters. Adsorption isotherms revealed that type I had larger maximal monomer and dimer capacities than type II, due to an electrostatic process. Mixture adsorption prefers dimers over monomers, with average values of 4.3 and 5.8 for type I and type II, respectively. The dynamics of competitive binding were heavily impacted by pore diffusion.^121^ Another study discusses the use of gradient elution chromatography with CHT columns to separate monoclonal antibody-monomer-dimer mixtures. The focus is on separation under overload conditions with phosphate gradients. Phosphate gradients are more effective than sodium chloride gradients and result in high yield and purity. The study presents a hybrid model based on empirical descriptions and stoichiometric shifting to optimize the operating conditions. The model fits experiments well and helps understand limiting factors.^122^

## Engineered fragmented monoclonal antibodies

###  Affinity chromatography (AF)

 Affinity chromatography is also performed in the purification process of modified mAbs. Protein G affinity chromatography is used to obtain a substantially pure scFv-Fc fusion protein, although there may be some smaller co-purifying bands that represent breakdown products binding to protein G.^123^ Epitope affinity chromatography effectively purifies mAbs and recombinant fragments. In 2002, researchers successfully isolated and purified these antibodies and fragments. They then conducted biophysical analyses to explore their properties. The study focused on evaluating the binding affinity and stability of the purified antibodies and fragments, which are critical for diagnostic and therapeutic applications.^12^ A 2010 study examined how the structure of human IgG mAbs affects their purification retention time using hydroxyapatite chromatography with sodium chloride gradient elution. The research involved 37 recombinants human mAbs to investigate the relationship between antibody structure (light chain subclass and type) and retention time. The results suggest that retention time is influenced by two primary interaction types: phosphoryl cation exchange and metal affinity interactions. Metal affinity interactions have a stronger influence on the gradient elution of sodium chloride than on the gradient elution of sodium phosphate. Recognizing these interactions is critical for refining the purification process to effectively isolate and purify the target mAbs.^124^

###  Hydrophobic interaction membrane chromatography 

 In 2014, a research study evaluated protein-A affinity chromatography against HIMC for the purification of a humanized heavy-chain monoclonal antibody (EG2-hFc). The findings indicated that samples of EG2-hFc purified by both methods exhibited high purity and comparable glycan profiles. This implies that HIMC might serve as an effective single-step technique for purifying the antibody while maintaining elevated purity levels and glycosylation patterns akin to those achieved with protein-A affinity chromatography.^94^ A 2015 review article examined protein L affinity chromatography for the purification of antibody fragments and highlighted its effectiveness in isolating and purifying scFv from complex mixtures. However, peptide tags can cause problems in the folding, association, degradation, and aggregation of scFv fragments. Protein L provides a superior approach to purifying histidine-tagged scFv fragments.^85^

 A 2016 study examines the purification of scFv using pH gradient SMB chromatography. The new method uses an open 3-zone pH gradient for continuous operation that attracts metal ions for separation. This technique modulates scFv adsorption and desorption, resulting in good purity and recovery, with empirical evidence supporting its effectiveness.^125^

 Overall, these studies show that various chromatography methods are successful in purifying monoclonal antibody fragments, underlining the need to choose the right approach depending on the fragments’ features and requirements. Furthermore, the use of IEX, SEC-MS, HIC, and HIMC yields useful information about the properties of mAb fragments. Furthermore, multimodal chromatography is a potential method for high-throughput purification.

###  Multicolumn counter-current solvent gradient purification (MCSGP) method

 Due to the presence of some challenges in the separation of charge variants of mAbs through conventional chromatography techniques, a more advanced process design known as MCSGP is introduced.^126^ Multicolumn Countercurrent Solvent Gradient Purification is a chromatography method developed by Aumann and Morbidelli at the Swiss Federal Institute of Technology Zürich for purifying biomolecules like mAbs from complex mixtures, using two to six connected columns.^126^ The MCSGP method has demonstrated its effectiveness in resolving the problem of overlapping elution profiles of proteins that pose a challenge in separation. This method provides enhanced purity and recovery and offers several advantages, such as achieving a high yield of desired purity, reducing buffer consumption, and improving productivity. ChromaCon holds a patent for this process, which is recognized for its user-friendly and uncomplicated design.^127^

 A study by Müller-Späth et al examined a two-step chromatography process for purifying mAbs. The process involves using cation exchange MCSGP as a capture step, followed by anion exchange in bind-elute mode for polishing. The developed 2-step process was compared to a 3-step process using protein A, anion exchange, and cation exchange. The performance of the 2-step process was similar in terms of purity, yield, productivity, and buffer consumption. The potential of the MCSGP process was also compared to a classical batch process using the same stationary phase.^128^

 Müller-Späth et al demonstrated that IEX could completely replace an expensive protein -A resin for mAb purification. Using a lumped kinetic model with linear batch gradient delusions and an experimental setup, they demonstrated that cation-exchange chromatography in a MCSGP system could produce 25 g mAb with up to 97% purity.^129^ Due to the trade-off between yield and purity in single-column batch chromatography, an article in 2020 discussed the utilization of continuous chromatography for bio-therapeutics. Their focus was on MCSGP to improve this trade-off. MCSGP allows the recycling of impure fractions, increasing yield without compromising purity. However, challenges remain, including robust models and automation.^130^

 In 2021, researchers investigated the new MCSGP approach to separate the charge variants of mAbs. The MCSGP method successfully separated charge fluctuations using cation and anion exchange chromatography with salt and pH gradients. As the data shows, CEX and AEX can isolate both charge variants, but with some differences, IEX with a pH gradient is best for acidic species, while CEX with a salt gradient is best for basic species. The MCSGP approach also improved the detection and characterization of post-translational changes.^131^ So MCSGP, a method that recycles overlapping components in mAb elution, has the potential to improve separation, but determining working details can be challenging due to variations in protein elution behavior.

 A study conducted in 2023 proposes an expanded design approach, referred to as a redesign for MCSGP, which involves changing the working specifications according to the elution profiles. Experimental results indicate that reMCSGP outperforms conventional MCSGP, achieving a recovery rate of 97.8% while maintaining purity above 95%, significantly outperforming standard MCSGP at 84%.^127^

 According to the studies reviewed, chromatographic methods are considered effective in purifying mAbs, and also some non-chromatographic methods represent valuable complementary approaches.

## Discussion and Conclusion

 The purification process of mAbs on the industrial scale usually includes various purification techniques, each with a specific role in mAb isolation to ensure high-purity, safe, and efficient therapeutic products. Chromatographic techniques in mAb purification owing to their exceptional capability in separating mAbs based on their unique physicochemical properties, are predominant. As a key factor, affinity chromatography offers unmatched specificity in capturing mAbs by interacting with specific antigen-binding sites. This first capture step is critical to maintaining product purity and improving downstream processing efficiency, so this method is mentioned in the production process of many mAbs available in the pharmaceutical market. However, despite all these positive aspects, this method has limitations such as the possible leaching of the protein A resin and the need for careful elution conditions to maintain the integrity of the antibodies.^81^ In addition to the affinity chromatography method, techniques such as IEX and HIC are also complementary methods. Ion exchange chromatography (IEX) acts as a precise tool to separate mAbs based on their overall charge.^26^ It is important to note that IEX remains valuable in the purification of PEGylated mAbs, by using the difference in charge properties between native and PEGylated biomolecules.^132^

 Beyond the capture phase, the SEC in the intermediate purification step is essential, because it separates mAbs based on their size helping to remove aggregates, fragments, and other forms that can potentially compromise the quality and safety of the final product. In addition, it is used for buffer switching to transfer the final product to its respective buffer.^133^ Furthermore, HIC is crucial in intermediate or polishing steps, especially when producing mAbs using mammalian cells as host cells.^41,45^ Innovative mAb purification techniques lead to important economic and therapeutic advancements. Multimodal ligand membrane adsorbers or columns can combine multiple separation methods in just one unit. Therefore it could be expected that the mentioned continuous processing method can enhance efficiency and minimize processing time.^134^ Investigations and studies in the domain of increasing the purity of mAbs are currently being carried out.

 To select a set of chromatographic techniques for the purification of a monoclonal antibody, it is required to have complete information about the antibody characteristics (pI, size, binding affinity), impurity profile, production scale, and regulatory requirements. Antibodies with low binding affinity may require ion exchange as the primary method, while high levels of impurities such as proteins or host cell beads may require a mixture of affinity and ion exchange chromatography. Production scale can also influence the selection of method, it is noticeable that in large-scale monoclonal antibody purification, which is very cost-effective, the application of purity and safety standards are far more accurate than in smaller-scale processes. Therefore, in most of these processes that are carried out for the manufacturing of antibody products on a large scale, multi-stage purification processes are needed that integrate several chromatographic methods to achieve the required purity.^13,81^

 In the upcoming discussion, the practical application of these chromatography techniques will be studied using a case study based on the table of chromatography methods employed in the purification of several FDA-approved mAbs.

 For example, the monoclonal antibody tocilizumab’s purification process, used to manage and treat MS, involves a series of steps starting with affinity chromatography, followed by IEX and SEC methods (Table 1). In addition, factors such as optimization of buffer conditions and elution profiles should be investigated according to the specific properties of the antibody. This process results in a modest amount, and after this phase, additional purification methods such as IEX, HIC, and SEC are incorporated into the process. Depending on the physical-chemical properties and impurity profile, two or all three of these methods can be used.^135-137^

**Table 1:** Chromatographic strategies for monoclonal antibody purification: A historical perspective (1997- 2024)

**Approved year**	**Monoclonal antibody**	**Capture**	**Intermediate**	**Polishing**		**References**
1997	Rituximab (Rituxan)	Protein A	Anion/cation exchange	HIC	HP-SEC Mixed-mode	^138-140^
1998	Trastuzumab (Herceptin)	Protein A	Anion/cation exchange	HIC	HP-SEC Mixed-mode	^139,141^
2002	Adalimumab (Humira)	Protein A/G	Anion/cation exchange	HIC	HP-SEC Mixed-mode	^139,142^
2003	Tocilizumab (Actemra)	Protein A	Anion/cation exchange	HIC	Mixed-mode	^137^
2004	Cetuximab (Erbitux)	Protein A	Anion/cation exchange	-	-	^143,144^
2004	Bevacizumab (Avastin)	Protein A	Anion/cation exchange	-	-	^22,145^
2009	Golimumab (Simponi)	Protein A	Anion/cation exchange	-	-	^22^
2010	Denosumab (Prolia/Xgeva)	Protein A	Cation exchange/mixed mode	-	-	^146^
2012	Pertuzumab (Perjeta)	Protein A	Cation exchange	-	-	^147,148^
2013	Obinutuzumab (Gazyva)	protein A/ Ni-NTA	Cation exchange	-	-	^149,150^
2015	Nivolumab (Opdivo)	Protein A	Cation exchange	-	HP-SEC Mixed-mode	^151^
2016	Atezolizumab (Tecentriq)	Protein A/ G	Cation exchange	HILIC	UPLC-SEC	^25^
2017	Emicizumab (Hemlibra)	Protein G	Cation exchange	HILIC	SEC-MS	^152^
2018	Trastuzumab-dkst (Ogivri)	Affimer reagents	Cation exchange	HIC	SEC-MS	^40,153^
2020	Bamlanivimab (COVID-19 mAb)	protein A/ Ni-NTA	Anion/cation exchange	-	SEC	^154^
2021	Tixagevimab/Cilgavimab (Evusheld)	protein A	Anion/cation exchange	-	HP-SEC	^155,156^
2022	Lecanemab (Leqembi)	Protein A/ G	Cation exchange	HIC	SEC	^157,158^
2024	Talquetamab (Talvey)	protein A	Cation exchange	-	SEC	^159^

 Variation in the use of different methods (Table 1) may be an indication of the flexibility of these methods for the specific mAb required. To better understand the concept of flexibility, the purification procedure for rituximab, a chimeric anti-CD20 monoclonal antibody used to treat B-cell malignancies, could be taken into consideration for a better understanding.^160^ Several research investigations indicate that multiple methods were used during the manufacturing and purification phase of rituximab and its biosimilars, especially in the capture phase, with the following strategies divided into intermediate and polishing phases depending on this initial capture phase.^139^

 There are two different processes for purifying rituximab and its biosimilars. In the first process, protein A affinity chromatography is performed during the capture phase using the UNOsphere SUPra protein A affinity resin column. In the intermediate phase, the Nuvia-Q column is used for strong anion exchange chromatography, and in the final polishing-cleaning phase, the Nuvia C-Prime column is employed in the mixed-mode chromatography technique. The second purification procedure for the rituximab biosimilar is described as follows: The capture purification phase is significantly different from the first process, which includes the strong cation exchange technique and the Nuvia S column, while the intermediate stage corresponds to the previous approach, which uses strong anion exchange Nuvia -Q column and for the polishing step the CHTXT column is used in mixed-mode chromatography.^140^ According to the points mentioned, it can be concluded that although a suitable model for mAb purification can be designed, for specific purification for one special of mAbs, attention should be paid to factors such as antibody properties and purification standards. Depending on these features, it is sometimes necessary to make changes to the overall model of the purification process. Additionally, manufacturing decisions, including purification techniques, column types, and buffer selection, must be considered.

## Competing Interests

 There are neither ethical nor financial conflicts of interest involved in the manuscript. The manuscript was not submitted for publication elsewhere.

## Consent for Publication

 Not applicable.

## Ethical Approval

 Not applicable.
